# Arthroscopically guided navigation for repair of acromioclavicular joint dislocations: a safe technique with reduced intraoperative radiation exposure

**DOI:** 10.1186/s13037-015-0087-0

**Published:** 2015-12-22

**Authors:** Jan Theopold, Bastian Marquass, Nikolaus von Dercks, Maria Mütze, Ralf Henkelmann, Christoph Josten, Pierre Hepp

**Affiliations:** Department of Orthopaedics, Trauma and Plastic Surgery, University of Leipzig, Liebigstr. 20, 04103 Leipzig, Germany; Devision of Arthroscopy, Joint Surgery and Sport Injuries, University of Leipzig, Liebigstr. 20, 04103 Leipzig, Germany

**Keywords:** Navigation, AC joint injuries, AC joint dislocation, AC joint reconstruction, Shoulder, Coracoclavicular

## Abstract

**Background:**

Accuracy evaluation of navigated image free placement of double cortical fixation buttons for coracoclavicular tunnel position in comparison to conventional drill guide based placement.

**Methods:**

Twenty-six patients with acute acromioclavicular joint instability were included in this non-randomized cohort study. All patients were treated with a Double- TightRope technique. In 13 cases the conventional drill guide based placement was used (group 1). In 13 patients surgery was performed as a navigated procedure with a fluoro-free optoelectronic system (group 2). The number of coracoclavicular drillings per patient (First pass accuracy; FPA (%)) was documented, the subcoracoidal position of the fixation buttons has been evaluated and graded as “intended position achieved (IPA)” or “intended position not achieved (IPnA)”.

**Results:**

In group 1 drilling had to be repeated in four patients (30.8 %) to achieve proper placement of the subcoracoidal fixation buttons. 100 % first pass accuracy was observed in group 2 (*p* = 0.03). In group 1, the intended position of the subcoracoid buttons was not achieved (IPnA) in six patients (46.2 %). In group 2 all intended positions were achieved (*p* = 0.005).

**Conclusion:**

Arthroscopic controlled fluoro-free navigated coracoclavicular drilling for the repair of acromioclavicular joint dislocation has higher first pass accuracy in comparison to conventional drill guide based placement. Therefore the navigation enables a precise position of the drill holes, may reduce the risk of an iatrogenic coracoid fracture and migration of fixation devices.

**Trial registration:**

Local institutional review board No. 061-14-10032014

## Background

Reconstruction of the coracoclavicular ligaments after acute acromioclavicular dislocation functions to restore anatomic alignment of the clavicle, may improve biomechanical stability and clinical outcomes [1–5]. Arthroscopic assisted procedures for transclavicular-transcoracoidal drilling and the use of cortical fixation buttons armoured with synthetic or autologous augmentation material have arisen and were developed to enable proper placement.

Improper placement of the buttons may lead to persisting instability, loss of reduction, coracoid fracture and slip of the coracoid button with subsequent recurrent vertical instability [6–10]. Taking into account anatomical variations of the coracoid, clavicle and the patient’s individual soft tissue proportion around the shoulder, one may agree that the intended position is not always achieved with a rigid drill guide in arthroscopic assisted placement of the tunnels. Therefore methods of fluoro free navigated coracoclavicular drilling have been developed to avoid repeated drilling and thus weaken the bone [11–14]. These procedures represent a simplified approach to navigating different instruments in relation to visually or tactilely placed pointers or objects without the need for radiation exposure or invasive fixation of a dynamic reference base in the bone [15]. As the feasibility and accuracy of fluoro free demonstrated in cadaver studies [14, 16, 17], clinical application has not been reported, yet.

Therefore the purpose of this retrospective case control study was to evaluate the accuracy of arthroscopic controlled navigated placement in comparison to conventional drill guide based placement of two coracoclavicular tunnels for double cortical fixation buttons in a clinical setup.

## Methods

This retrospective, non-randomised cohort study was approved by the local institutional review board (Commission of ethics, Medical Faculty, University of Leipzig, No. 061-14-10032014). The patients were informed about risks and benefits of the surgical technique and apprised that their data could be used for research. All patients gave their written informed consent before undergoing the operation.

### Patients

Twenty-six consecutive patients with acute acromioclavicular joint separation were treated with a Double-TightRope technique using the second-generation implant. Thirteen patients (0 female/13 male) with a mean age of 38 years (range 24–49 years) were treated using the arthroscopic assisted standard technique with drill-guide based placement of the two drill holes (group 1). Thirteen patients (one female/12 male) with a mean age of 38 years (range 21–56 years) therapy was supported by the arthroscopic controlled navigation of the coracoclavicular tunnels (group 2).

Injuries occurred predominantly during cycling/moto-cycling accidents (*n* = 16), alpine sports (*n* = 6) and other trauma (*n* = 4). In group 1, injuries were classified as Rockwood III injuries (*n* = 1, with a horizontal instability), Rockwood IV (*n* = 2) and Rockwood V (*n* = 10) injuries. Group 2 showed a comparable distribution of injuries: Rockwood III injuries (*n* = 2, both with horizontal instability), Rockwood IV (*n* = 2) and Rockwood V (*n* = 9) injuries.

### Surgical technique

The technique of arthroscopic assisted stabilization of acute acromioclavicular joint separations with double cortical fixation buttons has been described previously [5, 6, 18]. The basic principles comprise a standard diagnostic arthroscopic surgery in general anaesthesia and beach chair position. In cases of glenohumeral concomitant lesions, those were treated first. Then, an anteroinferior working portal just above the subscapularis tendon as well as a lateral viewing portal was established using the outside-in technique. Next, the subcoracoidal space and the base of the coracoid were prepared with the aid of a radiofrequency ablation device introduced through the anteroinferior portal [18].

A 5 cm sagittal incision with respect to the Langer’s lines was made over the clavicle approximately 1.5 cm medial to the AC joint. Subcutaneous skin flaps were elevated, the underlining deltoidtrapezoid fascia was identified and a T-shaped incision of the fascia was performed over the AC joint and the lateral clavicle. The AC joint was disengaged from trapped parts of the AC capsule and AC ligaments. The articular disc was excised when severely damaged. After reduction of the AC joint an acromioclavicular K-wire was temporarily placed in order to keep the AC joint stable during the next steps. The correct reduction of the AC joint was evaluated with an image intensifier in all cases.

**Fig. 1:**
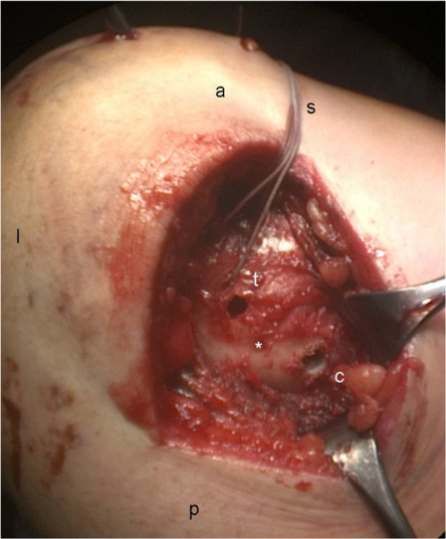
Clavicular footprint of the trapezoid (t) and conoid (c) ligament, * 20 % ratio of clavicle length. Left shoulder a: anterior, l: lateral; p: posterior; s: sutures for transosseous refixation of deltoid muscle detachment

In the course of this retrospective study the authors have changed their approach regarding the anatomical positions marking the clavicular and coracoid targets for coracoclavicular drilling. Whereas in the patient 1–14 the clavicular positions of the trapezoid and conoid were set at 20 and 40 mm from the lateral end of the clavicle, the clavicular locations of the CC ligaments in the subsequent patients 16–26 were determined as follows: One point at 20 % of the total clavicle length was set medial from the lateral clavicular edge and marked. This point approximately corresponds to the mean distance between the conoid and trapezoid clavicular footprint of 17 and 24 %, respectively [19]. With this point as a landmark, a dorsomedial (conoid) and an anterolateral (trapezoid) footprint region for clavicular drilling was defined, leaving a bony bridge of at least 5 mm to the posterior and anterior border of the clavicle, respectively. Unicortical drill holes (3.5 mm) were set at the clavicular footprint of the trapezoid and conoid ligament (Fig. 1).

**Fig. 2:**
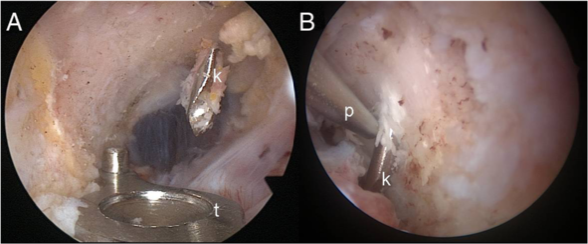
Arthroscopic subcoracoidal view. **a** k-wire placement (k) with drill guide (t). **b** navigated placement with the tip of the pointer (p) placed within den intended region

The targeted subcoracoidal regions changed, as well: whereas in the patient 1–14 the subcoracoidal positions of the trapezoid and conoid were positioned laterally and medially at the undersurface of the coracoid process close to its base in one line, the optimal subcoracoidal locations of the CC ligaments in the subsequent patients 15–26 were determined as follows: the target zone for the conoid coracoidal tunnel was at the posterior aspect of the coracoidal base, 5 mm lateral to the medial border. The aim for the trapezoid coracoid tunnel was 10 mm anterior to the conoidal tunnel and 5 mm medial to the lateral border of the coracoid, leaving a bony bridge between tunnels of at least 10 mm [5, 20] (Fig. 2).

### Drill guide procedure (group 1)

An aiming device for drilling (Constant Guide for AC TightRope, Co. Arthrex, USA) was used and its tip positioned at the undersurface of the coracoid process close to its base through the anterior-inferior portal. The device’s counterpart was placed on top of the clavicular body. A 2.0-mm K-wire was introduced into the device’s drill sleeve and locked into the unicortical clavicular drill hole. This enabled a joystick-like handling of the drill guide without misplacing the anatomic starting point on top of the clavicle.

After arthroscopic placement of the marking hook in the subcoracoidal target area, the drill guide was held in this position while placing the K-wire through the clavicle and the coracoid for both drillings. The drilling was repeated, if the K-wire missed the coracoid or did not respect a distance of at least 5 mm to the lateral or medial border of the coracoidas well as the first tunnel. All other “non-intended” positions were accepted in order not to weaken the bone with additional drillings.

The K-wires were overdrilled using a cannulated drill bit (4.0 mm). A delivery device (Application Sleeve & Pusher, Co. Arthrex, USA) was then introduced into the coracoclavicular tunnels and the TightRope devices were pushed through the delivery device until the oval-shaped buttons flipped beneath the coracoid arch under arthroscopic control.

The surgeon pulled on the No. 5 Fiberwire sutures (Co. Arthrex, USA), thereby tensioning both TightRopes by alternating the pull between the two devices. The sutures were knotted.

The detached deltoid and trapezoid muscle was anatomically fixed to the lateral clavicle with transosseous sutures (No. 2 Fiberwire, Co. Arthrex, USA) and closure lumbrification of the deltotrapezoid facia was performed. The T-shaped incision over the AC joint was closed.

Finally the temporary acromioclavicular K-wire was removed under image intensifier control. The superior incision was closed in two layers and the arthroscopic portals in a standard fashion. Postoperatively, radiographs were taken in anterior-posterior and axillary views.

### Navigated procedure (group 2)

For the navigation procedure (Fig. 2b), an established optoelectronic system with a fluoro-free software module was used (Trauma 2D 3.1 software, Co. Brainlab, Germany). The technique has previously been described in a cadaver model [14].

**Fig. 3:**
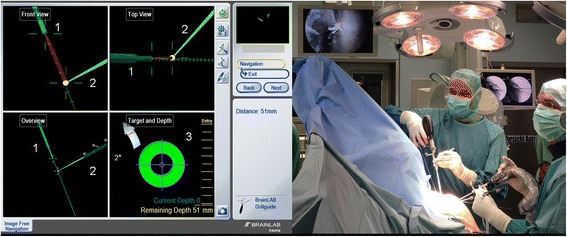
Virtual control of navigated drilling. **a** The navigation screen displays the instruments in three different views (*front, top and overview*) and the autopilot feature with online distance measurement (3) between the tips of instruments after exact instrument alignment; (1) navigated drill sleeve; (2) referenced pointer with the yellow ball representing the tip as target point. **b** Set up of arthroscopic controlled navigation

After calibration of the instruments, the tip of the pointer was positioned at the subcoracoidal target area through the anterior-inferior portal under arthroscopic control by the assistant (Fig. 3). The surgeon locked the drill sleeve into the unicortical clavicular hole of the conoid footprint and drilling of the 2.0 mm K-wire was performed through the clavicle and coracoid. The subcoracoidal perforation of the K-wire was visualised arthroscopically. The second transclavicular-transcoracoidal K-wire was established in same fashion. The further steps corresponded to the drill-guide based method (see above) (Fig. 3).

### Evaluation of first pass accuracy and subcoracoidal implant position

The number of coracoclavicular drillings per patient (first pass accuracy; FPA (%)) was documented.

**Fig. 4:**
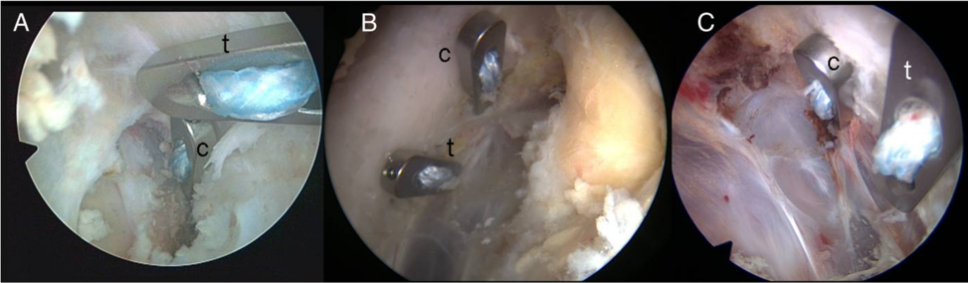
Subcoracoid button configuration; **a**: parallel configuration (*left shoulder*) **b**: stepwise configuration with the trapezoid tunnel being 10 mm anterior to the conoid tunnel and 5 mm medial to the lateral border of the coracoid, leaving a bony bridge between tunnels of at least 10 mm (*right shoulder*) **c**: non intended mirror-inverted stepwise placement (*left shoulder*). c: button for conoid tunnel; t: button for trapezoid tunnel

The subcoracoidal position of the fixation buttons was evaluated on the intraoperative images and movies (AIDA Compact III Neo, Storz, Germany) and graded as “intended position achieved (IPA)” or “intended position not achieved (IPnA)”. As mentioned above, the intended position for patients 1–15 was an almost parallel configuration whereas the intended position for patient 16–26 was a steplike placement of the subcoracoidal buttons [5] (Fig. 4).

**Fig. 5:**
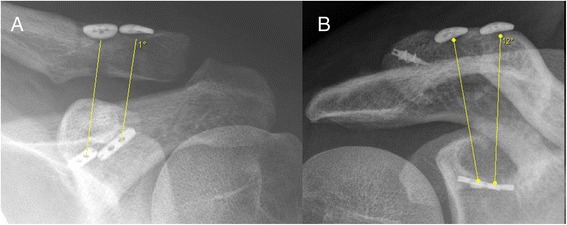
Measurement of the angle between trapezoid and conoid tunnel. **a**: left shoulder from a patient after drill guide based drilling with parallel tunnel configuration and **b** right shoulder from a patient after navigated drilling with v-shaped tunnel configuration

 The angle between the trapezoid and conoid drill tunnelswas measured on postoperative anterior-posterior radiographs of the shoulder (MagicWeb Software, version VA60C_0212, Visage Imaging, Germany) (Fig. 5).

#### Statistics

Results are expressed as mean ± standard error. For statistical analysis, SPSS (version 20, SPSS Inc., Chicago, USA) was used. The Kolmogorov–Smirnov test was used on all data to test for normal distribution. Student’s *T*-test was performed in cases of normal distribution. Chi square test was used to test differences between the numbers of drilling in each group. The significance level was set to *p* < 0.05.

## Results

### First pass accuracy

In group 1, drilling had to be repeated in four patients (4/13; 30.8 %): Three additional drillings were performed in one patient to achieve proper K-wire placement, two additional drillings in one injured and each one additional drilling in two patients, resulting in 33 drillings. In group 2 a 100 % (13/13) first pass accuracy was observed (*p* = 0.03).

### Position of the subcoracoidal buttons

In group 1, the intended position of the subcoracoid buttons was not achieved (IPnA) in six patients (6/13; 46.2 %). Four patients with “IPnA” showed steplike configuration and two patients showed a mirror-inverted placement of the subcoracoidal buttons (Fig. 4c). In group 2 all intended positions were achieved (IPA; 100 %; *p* = 0.005; Table 1).

**Table 1:** Patient data

Patient no.	Sex	Age	Rockwood Type	Placement	Intended position	Achieved position	K-wire correction (number of)	Concomitant lesions
1	m	30	5	C	parallel	parallel	2×	
2	m	49	4	C	parallel	m.-i. steplike	-	Pulley type 1
3	m	37	5	C	parallel	parallel	-	Bankart lesion
4	m	44	3	C	parallel	parallel	-	
5	m	35	5	C	parallel	parallel	1×	
6	m	41	5	C	parallel	parallel	-	SLAP 1
7	m	43	5	C	parallel	parallel	-	SLAP 1
8	m	32	5	C	parallel	steplike	-	
9	m	35	5	C	parallel	steplike	-	Labrumlesion, partial tear IST
10	m	48	4	C	parallel		3×	Partial tear SST
11	m	32	5	C	parallel	steplike	-	
12	m	46	5	C	parallel	m.i. steplike	-	
13	f	27	4	N	parallel	parallel	-	Partial tear SST
14	m	27	5	N	parallel	parallel	-	Pulley type 1
15	m	24	5	C	steplike	steplike	1×	
16	m	41	5	N	steplike	steplike	-	Pulley type 1
17	m	51	5	N	steplike	steplike	-	
18	m	56	3b	N	steplike	steplike	-	
19	m	47	5	N	steplike	steplike	-	
20	m	21	4	N	steplike	steplike	-	Fracture of the clavicle
21	m	41	3b	N	steplike	steplike	-	
22	m	29	5	N	steplike	steplike	-	
23	m	21	5	N	steplike	steplike	-	
24	m	35	5	N	steplike	steplike	-	SLAP 2
25	m	54	5	N	steplike	steplike	-	Partial tear SST
26	m	40	5	N	steplike	steplike	-	

*C* drill guide based drilling, *N* navigated drilling, *m.i*. mirror-inverted, *SST* supraspinatus tendon, *IST* infraspinatus tendon

### Trajectory of the coracoclavicular tunnels

The mean angles of both coracoclavicular tunnels were 7.7° (±4.1) in group 1 and 13.8° (±5.5) in group 2 (*p* = 0.007).

### Concomitant extra- and intraarticular lesions

One patient suffered an ipsilateral clavicle fracture, which was treated by open reduction and plate osteosynthesis. There were 11 concomitant intraarticular lesions (42.3 %): two type I superior labrum, anterior and posterior (SLAP) lesions, which were arthroscopically debrided, one type II SLAP lesion, which was repaired; three pulley type 1 lesions, which were debrided and shrinked with the aid of radiofrequency, three partial articular supraspinatus lesions (Elmann Typ A1), which were debrided, one PASTA lesion type A2, which was repaired and one patient with concomitant shoulder dislocation and a Bankart lesion, which was repaired. These repairs were performed arthroscopically.

### Intraoperative complications

One intraoperative complication comprised the conversion from the intended double cortical button fixation to a single button and additional hookplate fixation. Three additional K-wire drillings were performed until acceptable subcoracoid placement was achieved. Finally the conoid tunnel breached into the trapezoid tunnel when pulling on the TightRope device (patient no. 10, Table 1). The second cortical button had to be removed. A hookplate was then used due to the multiple drilling of the coracoid and the risk of subsequent coracoid fracture.

## Discussion

In this study, arthroscopic controlled navigated placement of two coracoclavicular tunnels for double cortical fixation buttons had a higher accuracy in comparison to conventional drill guide based placement. It is the first study reporting fluoro-free navigated transclavicular-transcoracoid drilling for double TightRope fixation in a clinical setup.

Coracoid and clavicle fractures remain a significant complication that occur predominately in techniques utilizing bone tunnel [21]. A correct placement of transclavicular-transcoracoidal tunnels may reduce the risk of repair failure and cortical breach, as emphasized in recent anatomic considerations for coracoclavicular ligament repair [7, 22, 23]. Although Mazzocca et al. [3] presented biomechanical results to support a more anatomic approach to the reconstruction of AC dislocations, others [19, 20, 24] carefully characterized the footprint of the CC ligaments in order to define the ideal location of ligament reconstructions. Not sufficiently known is first-pass accuracy of tunnel placement, especially for arthroscopically assisted procedures. It has been accentuated that transclavicular-transcoracoid drilling should be approached with caution [22].

Two studies analysing image-based navigated procedures for the placement of transclavicular-transcoracoid tunnels have been published [25, 26] showing a significant higher precision of navigation based placement when compared to conventional drilling under laboratory conditions [26] and in a clinical setup [25]. In both image based procedures the patients and specimen were exposed to a radiation time of 90 [25] and 60 s [26], respectively. Therefore, Hoffmann et al. emphasised that every effort must be made to minimise radiation exposure [16]. It was the first published fluoro-free arthroscopic assisted navigation procedure for coracoclavicular drilling, based on an electromagnetic system. The cadaver study described a 100 % successful tunnel placement and first pass accuracy in all cases. In a recent study Hoffmann et al. compared the navigation to the conventional drill guide based drilling for a double tunnel technique. The results showed a successful tunnel placement in 98.8 % compared to 83.8 % in the non-navigated group [17].

As the clavicular footprint is well defined [19] the subcoracoidal exit point is rather approximately described [24]. A real anatomic placement is not feasible and would lead to cortical breach as recently demonstrated [22, 23]. Therefore a near anatomic placement should be sought leaving a sufficient cortical bridge between the holes and the medial and lateral border of the coracoid. Anatomic references were utilized for the starting and exit point of the drill hole trajectory in the present study. The placement of the K-wires was performed without fluoroscopy. We used a clavicular starting point at 20 % of the total clavicle length as a reference point for both drill holes. This region approximatively corresponds to the mean distance between conoid and trapezoid footprint of 17 and 24 %, respectively [19].

### Limitations

Our study also has the same inherent weakness that is seen in many other retrospective studies. One concern may be the change of approach of the anatomical positions marking the clavicular and coracoid targets for coracoclavicular drilling in the course of the study. Nevertheless, the choice of starting and ending point for coracoclavicular drilling had no influence on the study aim evaluating the accuracy of each drilling procedure. As a consequence, the navigated tunnels showed a more v-shaped configuration in comparison to the non-navigated tunnels. Nevertheless, Kraus et al. [27] reported no significant differences regarding clinical or radiologic results when comparing both tunnel configurations. Secondly, drilling accuracy was evaluated by descriptive means in the present study. Others analysed the accuracy with CT scans [17] according the regions as introduced by Fereira [7]. A postoperative CT scan was not performed in the current study. The definition of “intended position” of the subcoracoidal buttons may therefore be subjective. On the other hand it reflects the clinical approach of implant positioning and position control.

Furthermore, operation time is of major concern, when using navigation systems. As some patients showed concomitant lesion which were treated during the surgery, a standardized documentation of the navigation time during surgery was not performed. Nevertheless, Hoffmann et al. [16] showed in their laboratory study a significant shorter surgery duration in the navigated specimen when compared to drill guide based placement.

## Conclusion

The repair of acromioclavicular joint dislocations with double cortical fixation buttons through arthroscopic controlled fluoro-free navigated coracoclavicular drilling has a higher first pass accuracy in comparison to conventional drill guide based drilling. Navigation may enable a precise anatomic position of the drill holes and reduce the risk iatrogenic coracoid fracture. Ultimately, comparative long-term follow-up studies are required to delineate the advantages in clininal outcome.
